# Microbiomic Insights into Differential Snow Mold Severity in Winter Cereal Crops

**DOI:** 10.3390/jof12070496

**Published:** 2026-07-07

**Authors:** Ildar T. Sakhabutdinov, Inna B. Chastukhina, Egor A. Ryazanov, Konstantin R. Yamschikov, Mira L. Ponomareva, Vladimir Y. Gorshkov

**Affiliations:** 1 Kazan Institute of Biochemistry and Biophysics, Federal Research Center “Kazan Scientific Center of the Russian Academy of Sciences”, 420111 Kazan, Russia; teilszx@yandex.ru (I.T.S.);; 2 Tatar Scientific Research Institute of Agriculture, Federal Research Center “Kazan Scientific Center of the Russian Academy of Sciences”, 420111 Kazan, Russia

**Keywords:** plant microbiome, plant infectious diseases, winter cereal crops, snow mold, co-occurrence networks, hub taxa, biological plant disease control

## Abstract

Winter cereals, which are vital for global food security in temperate regions, face severe challenges during overwintering due to the development of snow mold—a complex disease caused by different microorganisms that combine phytopathogenicity with cold tolerance. Even within a single field plot, individual plants exhibit significant variation in snow mold severity. This natural variation was exploited to achieve the aim of the present study—the comparison of microbiomes of healthy and diseased plants of winter cereal crops (rye, triticale, and wheat) at the peak of snow mold manifestation to interpret differential disease severity through differences in plant-associated microbial communities and to obtain information necessary for the biological control of snow mold. Fungi of the genus *Herpotrichia* were implicated as novel candidate causal agents of snow mold in winter cereals. Variations in snow mold severity defy simple explanations tied solely to pathogen abundance or broad changes in overall microbial community composition. Instead, the most striking contrast between healthy and diseased plants was observed in the inferred candidate hub taxa, accompanied by marked changes in exploratory co-occurrence networks involving the candidate snow mold pathogens. These network alterations were crop-specific. Several key taxa were implicated as probable influencers of snow mold dynamics.

## 1. Introduction

The plant microbiome is increasingly regarded as not merely a collection of associated microorganisms, but as a functional extension of the host plant, collectively forming the holobiont [1,2]. This integrated system enhances plant resistance to abiotic and biotic stressors through microbial contributions to nutrient acquisition, phytohormone production, pathogen suppression, and the induction of systemic resistance [3]. Insights into these plant–microbe interactions not only deepen our understanding of plant adaptive mechanisms and microbial community functioning, but also provide a valuable source of microbial strains for developing biopesticides, biostimulants, and sustainable crop protection strategies.

Advances in high-throughput amplicon sequencing have substantially improved our ability to accurately determine microbial taxonomic composition, identify disease-causing pathogens, and uncover associations between specific taxa and plant phenotypes [3]. However, taxonomic composition alone often fails to fully explain host plant phenotypes, as these phenotypes depend not merely on the presence or abundance of individual microorganisms, but also on dynamic interactions among them. Such interactions are commonly inferred from co-occurrence network analysis [4,5,6,7,8], which distinguishes positive and negative association patterns that are usually operationally assigned to synergistic processes, such as cross-feeding or niche-sharing, or antagonistic processes, such as competition or direct inhibition. Both types of interactions contribute to network stability and plant health, with their balance shaping microbiome resilience and holobiont homeostasis [9,10]. Disruptions in this balance, such as those induced by abiotic stressors or pathogen ingress, can lead to dysbiosis and compromise plant health. Key roles in community interactions are played by the taxa with the highest connectivity degrees, known as hub taxa. These taxa interact with a large number of other microorganisms within the community and form the primary nodes of co-occurrence networks [6,8,11,12,13,14].

Despite the growing body of research on plant microbiomes and their role in plant fitness, microbiomes of winter cereals—crops that provide a substantial proportion of global caloric intake, particularly in regions with prolonged winters—remain severely understudied. In temperate climates with cold winters and persistent snow cover, winter cereals predominate, owing to their higher yield potential and agronomic advantages over spring-sown crops. These advantages include more efficient utilization of early spring soil moisture, avoidance of summer drought, and completion of sensitive development stages before the onset of peak heat and water stress [15,16]. However, the overwintering strategy exposes these crops to a major threat: snow mold disease, which can cause yield losses of up to 50% in epiphytotic years [17]. Snow mold is a collective term encompassing a group of diseases caused by phylogenetically distant fungi that share two key traits: phytopathogenicity, and psychrotolerance or psychrophily. The most known causal agents of snow mold worldwide include *Microdochium nivale* (pink snow mold), *Sclerotinia borealis* (snow scald), *Typhula ishikariensis* (speckled snow mold), and *T. incarnata* (gray snow mold) [18]. In the central regions of Russia, *M. nivale* is considered the dominant species associated with snow mold outbreaks [19,20]. Nevertheless, damage to winter cereals during overwintering can also be caused by other psychrotolerant phytopathogenic fungi [18]; for example, in the Volga region, *Leptosphaeria sclerotioides* (anamorph *Phoma sclerotioides*) has been strongly implicated as a snow mold pathogen of winter wheat [21,22].

Snow mold management remains challenging. Breeding for resistance is constrained by the scarcity of reliable genetic donors and the complex polygenic nature of tolerance [23]. Chemical control is greatly complicated by the fact that disease progression occurs beneath snow cover, allowing only preventive fungicide applications before snowfall, which substantially reduces their effectiveness, along with the emergence of resistance or reduced sensitivity of the pathogens to fungicides [17,24,25]. Agronomic practices provide only inconsistent and partial effects in reducing snow mold severity. In this context, biological control agents capable of functioning at low temperatures and forming stable interactions with winter cereals represent a promising strategy for protecting these crops against snow mold.

However, effective microbial agents for this purpose have not yet been identified or validated. To our knowledge, only one study has demonstrated in vitro antagonism of *Pseudomonas* strains against *M. nivale* [26], but their efficacy on infected plants, particularly under field conditions, remains unknown. Microbiome profiling holds strong potential to facilitate the targeted identification of such protective strains. In our previous work—the only microbiome-based study of snow mold to date—we compared snow mold pathocomplexes across different winter cereal crops and predicted a set of candidate microorganisms with potential suppressive activity against *M. nivale* [22]. The present study builds upon this foundation by optimizing a model system to investigate microbiome-wide aspects of snow mold development and to lay the groundwork for biologically based disease management strategies for winter cereals. Notably, under moderate-to-moderately high snow mold pressure, even within a single field plot, individual plants exhibit marked heterogeneity in disease severity, ranging from complete plant death to virtually asymptomatic appearance. We exploited this natural variation to compare root microbiomes of plants with minimal (healthy) versus severe (diseased) snow mold symptoms.

Thus, the aim of the present study was to compare root microbiomes between healthy and snow mold-affected plants of winter crops (rye, triticale, and wheat) at the peak of disease manifestation, in order to explain differences in disease severity based on variations in plant-associated microbial communities and to generate insights relevant to the biological control of snow mold.

## 2. Materials and Methods

### 2.1. Experimental Design and Sample Collection

Microbiomes were analyzed in three winter cereal crops—winter rye, winter wheat, and winter triticale—using cultivars with moderate resistances to snow mold: winter rye (*Secale cereale* L. cv. Tantana), winter wheat (*Triticum aestivum* L. cv. Kazanskaya 560), and winter triticale (*×Triticosecale* Wittm. cv. Beta). Within each crop, plants were sampled at two contrasting levels of snow mold severity, hereafter referred to as “healthy” and “diseased”: healthy rye (6% (0–10%) damage, average disease score 2.3), diseased rye (78% (70–80%) damage, average disease score 6.6); healthy wheat (3% (0–10%) damage, average disease score 1.8), diseased wheat (54% damage (50–60%), average disease score 5.2); healthy triticale (5% (0–10%) damage, average disease score 2.2), diseased triticale (62% (60–70%) damage, average disease score 5.6). Disease severity was assessed using a 9-point scale, where 1 indicates no visible symptoms and 9 indicates complete plant death (100% damage) [23]. All crops were grown under uniform agronomic management in a single competitive variety field trial located in the Laishevo district of the Republic of Tatarstan, Bolshiye Kaban (latitude 55.625164 N, longitude 49.351334 E). No fungicides were applied at the experimental plots.

Plant samples were collected in 2021 (April 19) one week after snowmelt. Six experimental groups were analyzed (three crop species × two disease statuses). Each group consisted of 10 biological replicates, resulting in a total of 60 samples. Within each of the six experimental groups, plants were collected from two field plots (5 replicates per plot), with healthy and diseased individuals sampled from the same plots to ensure comparable environmental conditions. Whole plants were excavated in the field and transported to the laboratory at 4–5 °C. Roots were excised, washed thoroughly with distilled water to remove adhering soil, surface-sterilized in 70% ethanol for 10 s, and subsequently rinsed twice in sterile distilled water. The root samples were processed to primarily capture the endophytic microbial community, although a minor contribution of rhizoplane microorganisms cannot be excluded. The washed root samples were immediately frozen in liquid nitrogen and stored at −80 °C until further processing. All procedures from field collection to liquid nitrogen freezing were completed within one day; except, during washing, samples were kept at 4–5 °C throughout the procedure.

### 2.2. DNA Extraction, Library Preparation, and Sequencing

Total DNA was extracted from root samples using the DNeasy PowerBiofilm Kit (Qiagen, Hilden, Germany) following the manufacturer’s protocol. DNA quantity was assessed using a NanoDrop spectrophotometer (Implen, Munich, Germany). The fungal ITS2 region of the ribosomal RNA gene was amplified using primers ITS3_KYO2 (5′–GAT GAA GAA CGY AGY RAA–3′) and ITS4 (5′–TCC TCC GCT TAT TGA TAT GC–3′) [27]. The bacterial V3–V4 regions of the 16S rRNA gene were amplified using primers Bakt_341F (5′–CCT ACG GGN GGC WGC AG–3′) and Bakt_805R (5′–GAC TAC HVG GGT ATC TAA TCC–3′) [28].

Amplicon libraries were prepared according to the Illumina protocol (part no. 15044223, Rev. B). Indexing was performed using the Nextera XT Index Kit v2 (Illumina, San Diego, CA, USA). Libraries were sequenced on an Illumina MiSeq platform using the MiSeq Reagent Kit v3 (600 cycles) (Illumina). All datasets were deposited in the NCBI Sequence Read Archive (SRA) under BioProject accession PRJNA1380312.

### 2.3. Read Processing and Amplicon Sequence Variant (ASV) Inference

Raw reads were processed for quality control using FastQC [29] and MultiQC [30]. Primer sequences were removed using Cutadapt v3.5 [31]. The DADA2 pipeline [32] was employed for quality trimming, dereplication, filtering for chimeras, and inference of amplicon sequence variants (ASVs). For ITS2 libraries, forward and reverse reads were truncated at 230 and 190 bp, respectively (truncLen = c(230, 190)), and filtered using maximum expected error thresholds of 3 and 4, respectively (maxEE = c(3, 4)). For 16S rRNA libraries, forward and reverse reads were truncated at 270 and 220 bp, respectively (truncLen = c(270, 220)), and filtered using maximum expected error thresholds of 4 and 5, respectively (maxEE = c(4, 5)). Taxonomic assignment of ASVs was performed using a naive Bayes classifier trained on the UNITE v8.3 database for fungi [33] and the SILVA v138 database for bacteria [34]. Taxonomic annotations were manually refined and cross-checked against the NCBI Taxonomy, MycoBank, and Bacterio.net databases. Non-target ASVs (e.g., chloroplasts, plant nuclear/mitochondrial sequences, Metazoa) and low-abundance ASVs (constituting < 0.00001% of the total reads in fungal or bacterial datasets) were removed from downstream analysis [35]. Good’s coverage was calculated using MicrobiomeAnalyst [36] to assess sequencing depth and saturation. ASV sequences, abundance tables, taxonomic annotations, and sample metadata are available on GitHub at https://github.com/ildar-ITS/2026_Healthy_Diseased_16S_ITS2_Roots_W_T_R_microbiome (accessed on 27 February 2026).

### 2.4. Analysis of α- and β-Diversity and Identification of Differentially Abundant Taxa

Analysis of α- and β-diversity and identification of differentially abundant taxa (DAT) was conducted in R (v. 4.5.0) [37] using the ‘phyloseq’ package (v. 1.52.0) [38]. Data normalization was performed using the Cumulative Sum Scaling (CSS) method [39] implemented in the metagenomeSeq package (v. 1.50.0) [40], and CSS-normalized relative taxon abundances were visualized using microViz [41]. To test for significant differences in the CSS-normalized abundance of target phytopathogens (*Herpotrichia*, *Leptosphaeria*, *Microdochium*) between healthy and diseased crops, the Wilcoxon rank-sum test was applied using the rstatix package (v. 0.7.2) [42], with results visualized using the ggpubr package (v. 0.6.1) [43].

α-Diversity was assessed by calculating the Chao1, Shannon, and Gini–Simpson indices using the microbiome package (v. 1.30.0) [44]. Differences in α-diversity indices between experimental groups (crop × disease status) were tested with the Kruskal–Wallis test from the stats package and a post hoc Dunn’s test with Benjamini–Hochberg (BH) FDR correction using rstatix [42].

For β-diversity analysis, filtered raw ASV counts, after the removal of non-target and low-abundance ASVs, were used as the input. Principal Coordinate Analysis (PCoA), based on the robust Aitchison (rALR) distance, was calculated with the microViz package (v. 0.13.0) [41]. The significance of differences between groups was assessed by PERMANOVA and pairwise ANOSIM using functions from vegan (v. 2.7-5) [45], or veganEx (v. 0.1.0) if pairwise multilevel comparisons were required [46]. DAT between healthy and diseased plants within each crop were identified using a negative binomial (NEGBIN) (Benjamini–Hochberg FDR-adjusted *p* < 0.05) using MicrobiomeAnalyst (v.2.0) [36].

For all statistical analyses, individual plants were treated as plant-level biological replicates. Field plot identity was not explicitly included as a blocking or random factor; therefore, the results should be interpreted as plant-level associations within the sampled plots rather than as fully replicated field-level effects.

### 2.5. Network Construction, Correlation Analysis, and Visualization

To construct co-occurrence networks of the total fungal–bacterial communities, 16S rRNA library sizes were scaled to match the ITS2 library sizes for each sample, yielding a merged fungal–bacterial dataset. This merged dataset was used as an exploratory cross-domain co-occurrence framework to identify associations between putative fungal snow mold pathogens and bacterial community members. Such joint analysis enables the detection of interkingdom associations and hub structures that may be missed when bacterial and fungal communities are analyzed separately as single-domain networks [4,7,8]. Interkingdom edges inferred from the merged dataset are interpreted as exploratory co-occurrence associations rather than validated fungal–bacterial interactions. Using the NetCoMi package (v. 1.2.0) [47], co-occurrence networks were constructed at the genus level based on abundance correlations inferred with the SpiecEasi algorithm [48]. Taxa with a prevalence below 50% within the dataset for each crop type were excluded from the analysis. Networks were constructed using the netConstruct function, and network measures were obtained using the netAnalyze function. Network sparsity in SPIEC-EASI was selected using StARS stability selection. The model-selection output was inspected to confirm that stable network solutions were obtained before downstream analysis. Network measures were compared between the communities of healthy and diseased plants within each crop using the netCompare function with 1000 permutations. Hub taxa in each network were identified as the top 5% of taxa with the highest eigenvector centrality scores [6,8]. Networks were visualized using the interactive platform Gephi (v. 0.10.1) [49].

To identify fungal taxa whose relative abundance correlated with that of the target fungal phytopathogens (*Herpotrichia*, *Microdochium*, *Leptosphaeria*), genera with a prevalence below 25% were first filtered out. Spearman’s rank correlation coefficients were then calculated using the Hmisc package (v. 5.2-2) [50] separately for each crop and health status (resulting in six independent datasets). Only correlations with r > |0.5| and Benjamini–Hochberg FDR-adjusted *p* < 0.05 were considered. Bacterial genera whose relative abundance correlated with that of the target fungal phytopathogens were identified in a similar manner, using the merged fungal–bacterial dataset as input for the analysis.

## 3. Results

### 3.1. Most Abundant Fungal and Bacterial Taxa Inhabiting Roots of Winter Cereal Crops with Different Levels of Damage After Wintering

Information about the sequencing depth and numbers of the revealed amplicon sequence variants (ASV) and taxa is presented in Table 1 and Table S1.

**Figure 1:**
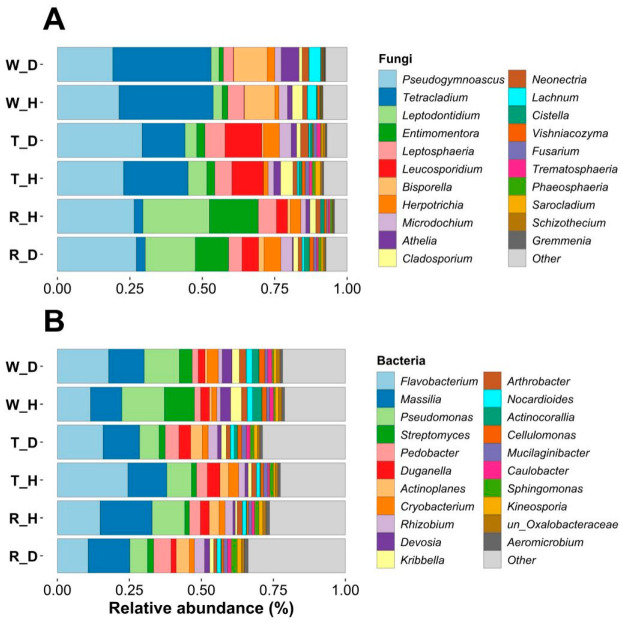
Cumulative Sum Scaling (CSS)-normalized relative abundance of the top 21 dominant fungal (**A**) and bacterial (**B**) genera in the root microbial communities of healthy (H) and diseased (D) plants across three winter cereal crops: wheat (W), triticale (T), and rye (R). The prefix “Un_” indicates unclassified taxa assigned only to a higher taxonomic rank (above genus).

Analysis of the taxonomic composition at the genus level revealed a high degree of similarity in the dominant microbial taxa associated with the roots of different winter crops (rye, wheat, and triticale), regardless of whether the plants were severely damaged after wintering (hereafter referred to as diseased plants) or exhibited minimal damage (hereafter referred to as healthy plants) (Figure 1).

The majority of the dominant genera comprise non-phytopathogenic microorganisms, which are unlikely to be responsible for the observed plant damage in diseased plants. Herewith, among the top 10 most abundant genera, the well-known causal agent of snow mold disease, *Microdochium*, was identified. Additionally, *Leptosphaeria*, ranked sixth, has been previously proposed as a candidate causal agent of snow mold [21,22,51,52,53]. Furthermore, the eighth-ranked genus, *Herpotrichia*, has not been previously reported to parasitize cereal crops, but has demonstrated psychrotolerant and phytopathogenic properties [18,54,55,56,57,58]. Other well-known snow mold pathogens, fungi of the *Sclerotinia* and *Typhula* genera, were not among the most abundant taxa and were present only at low levels (0.022% and 0.075%, respectively). Therefore, *Microdochium*, *Leptosphaeria*, and *Herpotrichia* were further evaluated in our study as primary candidates responsible for the observed plant damage.

**Figure 2:**
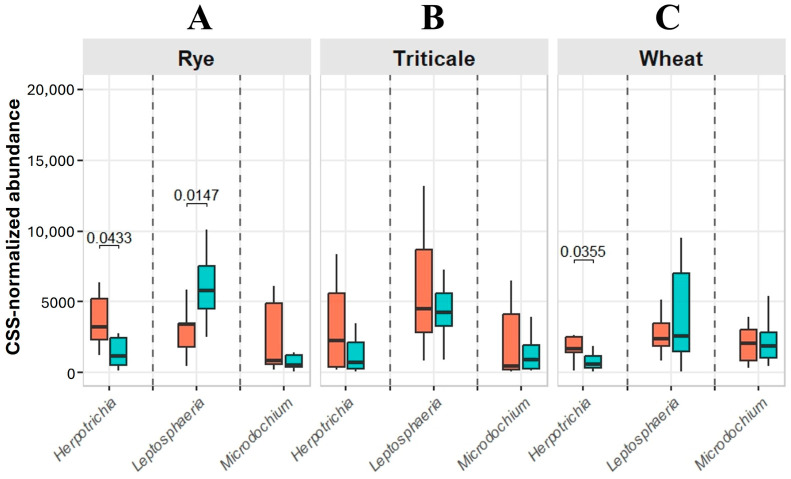
Cumulative Sum Scaling (CSS)-normalized abundance of three fungal genera (*Herpotrichia*, *Leptosphaeria*, and *Microdochium*) in the roots of diseased (red box plots) and healthy (green box plots) plants of three winter cereal crops: rye (**A**), triticale (**B**), and wheat (**C**). Significant differences in the abundance of these genera between healthy and diseased plants are indicated by brackets with corresponding *p*-values from targeted pairwise Wilcoxon rank-sum tests (*p* < 0.05; performed separately for each crop and each fungal genus).

The relative abundance of *Microdochium* and *Leptosphaeria* was not higher in diseased plants than in healthy plants (Figure 2). Furthermore, the abundance of *Leptosphaeria* was significantly higher in healthy rye than in diseased rye. In contrast, *Herpotrichia* was significantly more abundant in diseased rye and wheat than in their healthy counterparts (Figure 2).

### 3.2. α- and β-Diversity of Fungal and Bacterial Communities in the Roots of Winter Cereal Crops with Different Levels of Damage After Wintering

No significant differences were observed in the three α-diversity indices (Chao1, Shannon, and Gini–Simpson) between healthy and diseased plants for any crop, except that the Shannon and Gini–Simpson indices for fungal communities were significantly higher in diseased rye compared to healthy rye (Table S2). In contrast, some differences in α-diversity indices were found between different crops within a particular disease status (healthy or diseased) (Table S2).

**Figure 3:**
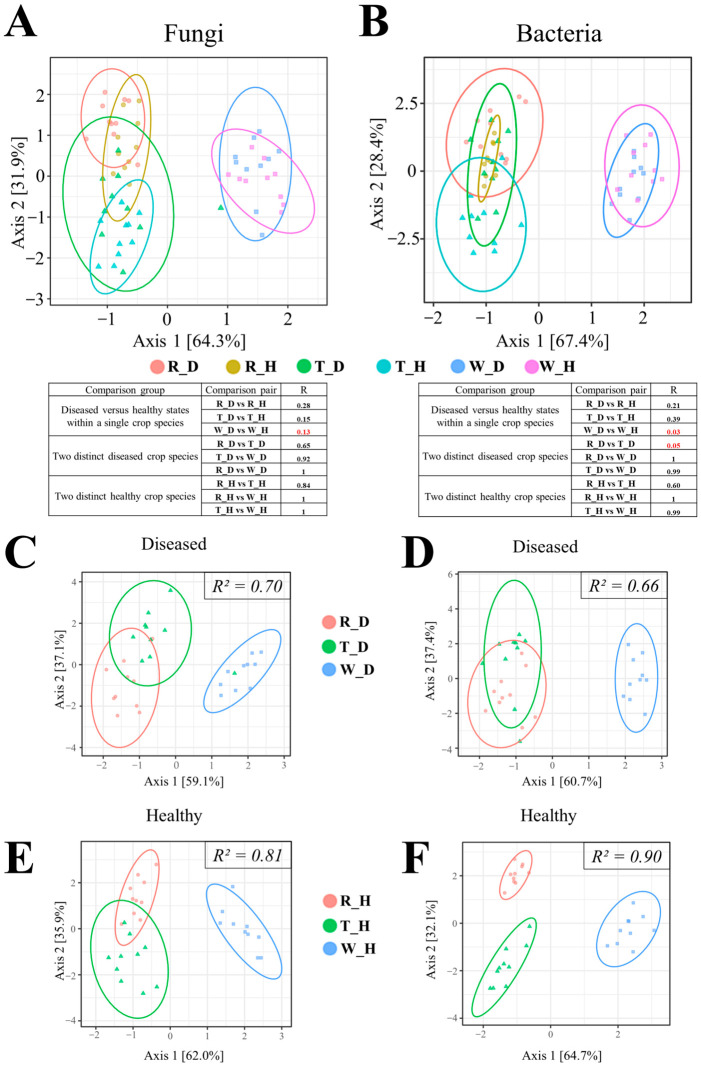
Principal Coordinate Analysis (PCoA) based on the robust Aitchison distance (rALR) of fungal and bacterial communities in the roots of healthy (H) and diseased (D) plants across three winter cereal crops: rye (R), wheat (W), and triticale (T). Panels (**A**,**B**) display the comparison of fungal (**A**) and bacterial (**B**) communities, respectively, for all six datasets combined (three crops, each with two disease statuses). The ellipses, representing 95% confidence intervals, are color-coded with the legend provided at the bottom of the PCoA plots. Tables below the PCoA plots show R-values (ANOSIM test, *p* < 0.05) for pairwise microbiome comparisons: communities of the same crop with different disease statuses (healthy vs. diseased) (3 pairs of comparisons); communities of different crops with the disease status “diseased” (3 pairs of comparisons); and communities of different crops with the disease status “healthy” (3 pairs of comparisons). Black and red values in the tables indicate significant (black) and insignificant (red) differences (ANOSIM, *p* < 0.05) within a comparison pair. Panels (**C**–**F**) show the comparisons of fungal (**C**,**E**) and bacterial (**D**,**F**) communities of the three crops separately for diseased (**C**,**D**) and healthy (**E**,**F**) plants. The R^2^ value in the upper right corner of each PCoA plot reflects the variability assessed by PERMANOVA for the triple comparison.

Differences in the β-diversity of both fungal and bacterial communities were mostly determined by crop species rather than disease status (Figure 3). In PCoA plots, fungal communities of wheat formed distinct clusters that did not overlap with those of rye and triticale communities, whereas the clusters corresponding to rye and triticale communities largely overlapped with each other.

ANOSIM tests confirmed the pattern observed in the PCoA plots: pairwise comparisons between different crop species yielded higher R-values than comparisons between healthy and diseased states within a single crop. ANOSIM tests did not reveal significant differences in community structures between healthy and diseased wheat, whereas the community structures of rye and triticale significantly differed between healthy and diseased plants. A similar trend in β-diversity differences was observed for bacterial communities (Figure 3). A triple comparison of crops within a particular disease status showed that, for fungal communities, between-crop variability was higher in healthy plants (R^2^ = 0.81) than in diseased plants (R^2^ = 0.70). For bacterial communities, this trend was even more pronounced: between-crop variability was 0.90 in healthy plants and 0.66 in diseased plants (Figure 3).

### 3.3. Microbial Taxa with Differential Abundance in Disease and Healthy Plants

To identify the specific taxa driving the observed differences in β-diversity between the microbiomes of healthy and diseased plants, a negative binomial distribution (NEGBIN) analysis was performed. This analysis identified taxa with differential abundance in diseased and healthy plants (DAT) across rye, wheat, and triticale. Among the DAT, both fungi (7–14 per crop) and bacteria (8–17 per crop) were revealed (Table S3). The relative abundance of many of these taxa was low (below 1%), which explains the relatively small differences in β-diversity between healthy and diseased plants.

Among the DAT, those with a relative abundance above 1% included the following genera: (1) within rye, the bacterial genera *Devosia*, *Kribbella*, and *Pantoea* were enriched in diseased plants, while *Luteolibacter* and *Janthinobacterium* were enriched in healthy plants; (2) within triticale, the fungal genus *Tetracladium* was enriched in healthy plants, whereas several bacterial genera (*Kineosporia*, *Caulobacter*, and *Actinocorallia*) were enriched in diseased plants; (3) within wheat, the fungal genus *Cladosporium* and the bacterial genus *Streptomyces* were enriched in healthy plants compared to diseased plants. Among these taxa, the highest relative abundance was observed for *Tetracladium* (21.9%), *Streptomyces* (9.40%), and *Cladosporium* (4.24%), all of which were more abundant in healthy plants compared to diseased plants. The relative abundance of taxa enriched in diseased plants was lower.

### 3.4. Correlation Networks Within Root Communities of Winter Cereal Crops with Different Levels of Damage After Wintering

To compare microbial networks in healthy versus diseased plants, exploratory cross-domain co-occurrence networks were constructed for the combined fungal and bacterial communities of wheat, rye, and triticale. The main topological properties of these networks are summarized in Table S4. The analysis of topological parameters was performed on both the whole network and its largest connected component (LCC), with two datasets yielding consistent results.

Pairwise comparisons of the global network properties revealed some crop-specific significant differences between healthy and diseased plants. In rye communities, modularity and average path length were significantly higher in the networks of healthy plants compared to those of diseased plants, potentially indicating a more complex and organized structure in the former. Higher modularity is typically associated with greater compartmentalization of the microbial community, which can potentially restrict the spread of invading taxa across the network [9]. In wheat communities, networks of diseased plants exhibited a significantly higher clustering coefficient compared to healthy plants, suggesting the formation of denser local microbial clusters within diseased plant communities. In triticale communities, no significant differences in network parameters were found between healthy and diseased plants. Other parameters of global network properties, such as edge density, the relative size of the LCC, and the percentage of positive edges, did not show consistent differences between communities of healthy and diseased plants (Table S4).

Significant differences between the communities of healthy and diseased plants were most pronounced in the shift of key nodes (hubs), as indicated by the values of different Jaccard indices calculated for various centrality metrics (degree, betweenness, closeness, eigenvector centrality, and hub taxa). Specifically, the Jaccard indices for candidate hub taxa within rye and wheat communities were zero (*p* < 0.001), indicating no overlap between the candidate hub taxa in healthy and diseased plants. In contrast, the index for triticale communities was low but not statistically significant, suggesting a lesser overall shift in hub taxa between healthy and diseased plants compared to rye and wheat communities (Table S4).

### 3.5. Hub Taxa in Root Communities of Winter Cereal Crops with Different Levels of Damage After Wintering

To identify hub taxa in the communities of diseased and healthy plants, the top 5% of taxa with the highest eigenvector centrality values, reflecting the connectivity of a taxon while also accounting for the centrality of its connected neighbors, were selected within each of the six microbial networks (corresponding to the communities of three crops, each with two disease statuses). Pairwise comparisons within each crop confirmed no overlap between hub taxa in healthy and diseased plants for rye and wheat communities, as evidenced by null Jaccard indices for hub taxa (Section 3.4). In triticale communities, most candidate hub taxa did not match between healthy and diseased plants; however, some hub taxa (*Plantibacter* and *Lapillicoccus*) were shared between the two plant health statuses (Table S5).

**Figure 4:**
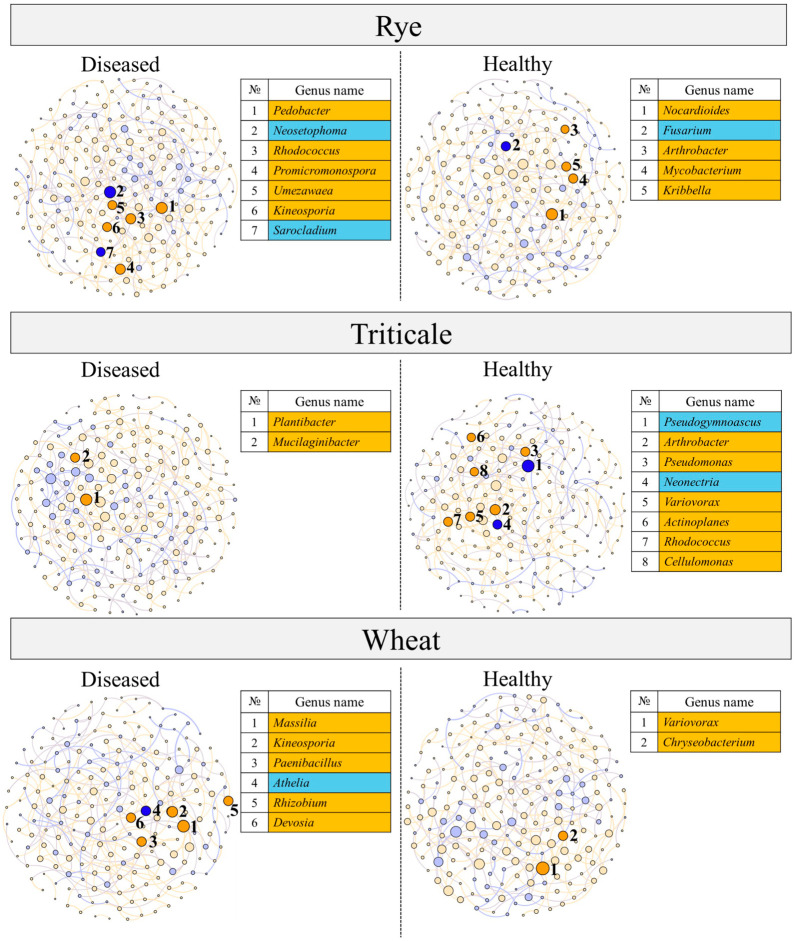
Correlation networks of root communities from healthy and diseased plants across three winter cereal crops: rye, triticale, and wheat. Circles represent nodes (taxa), while lines indicate co-occurrence associations between nodes. Node size is proportional to eigenvector centrality. Edge color indicates the type of association: blue edges represent fungal–fungal associations, yellow edges represent bacterial–bacterial associations, and gray edges represent fungal–bacterial associations. Nodes labeled with numbers correspond to key hub taxa that meet all three of the following criteria simultaneously: (1) belong to the top 5% of taxa with the highest centrality values in a particular network (hub taxa); (2) belong to core microbiome for a given sample (present in at least 80% of replicates); and (3) have an abundance above 0.1% within the community. Key hub taxa are indicated by numbers positioned to the right of the corresponding nodes in each network. Bacterial taxa are indicated by a yellow background, while fungal taxa are shown with a blue background. Key hub taxa in each of the six datasets are ranked by decreasing eigenvector centrality.

Approximately half of the identified hub taxa were either of low relative abundance within the communities (<0.1%) or did not constitute the core microbiome (i.e., were present in fewer than 80% of replicates within a given sample). To highlight the key hub taxa with the most expected effect on the community and the disease status of the host plant, only those hub taxa that both formed part of the core microbiome and had an abundance above 0.1% were selected. These key hub taxa are listed in Figure 4.

### 3.6. Taxa with the Most Expected Effect on Snow Mold Pathogens in Root Communities of Winter Cereal Crops with Different Levels of Damage After Wintering

Given the observed differences in hub taxa between the microbial communities of healthy and diseased plants, we hypothesized that local correlation links between the considered putative snow mold pathogens (*Herpotrichia*, *Leptosphaeria*, and *Microdochium*) and other community members would vary depending on disease status and that, depending on the nature of these links, the pathogenic potential of snow mold fungi could either be stimulated or repressed. To test this hypothesis, we performed Spearman rank correlation analyses between the abundance of snow mold pathogens and all other taxa within each of the six datasets (three crops, each at two disease statuses).

Only taxa with a relative abundance above 0.1% were considered. Taxa whose abundance showed a significant correlation (r > |0.5|, FDR-adjusted *p* < 0.05) with the abundance of at least one of the three snow mold phytopathogens were selected (Table S6). Among these, we highlighted taxa that met one or more of the following criteria: (1) belong to hub taxa (including key hub taxa); (2) are differentially abundant between healthy and diseased plants; (3) exhibit a reversal of correlation sign between healthy and diseased states (from positive to negative or vice versa). The taxa meeting these criteria (hereafter referred to as target taxa) are presented in Table 2.

The pattern of differences in target taxa between healthy and diseased plants was crop-specific (Table 2). In diseased rye, most target taxa (eight out of ten) were those whose abundances had positive correlations with the abundance of snow mold pathogens, whereas in healthy rye, only two target taxa were revealed, with the abundances of both showing negative correlations with the abundance of snow mold pathogens (Table 2). In wheat, six target taxa were found, with the abundance of five of them showing correlations with the abundance of *Herpotrichia*. The set of these five target taxa was identical between healthy and diseased plants; however, importantly, for all these taxa, the sign of the correlation was reversed between healthy and diseased plants. A similar trend was observed for only one target taxon, *Caulobacter*, within the rye community, and for only one target taxon, *Trematosphaeria*, within the triticale community. In triticale, the overall pattern of differences in target taxa between healthy and diseased plants was less pronounced than in rye (where a large number of target taxa showed positive correlations with snow mold pathogens in diseased (but not healthy) plants) and wheat (where target taxa exhibited reversed correlation signs between diseased and healthy plants) (Table 2).

## 4. Discussion

In this study, we examined the criteria that distinguish the microbiomes of plants with different levels of disease damage—minimal damage (healthy plants) versus severe damage (diseased plants)—to identify microbial markers of plant resistance or susceptibility. The study was conducted on the roots of winter cereal crops after overwintering, when snow mold disease reaches its peak. Snow mold is caused by psychrophilic and psychrotolerant fungi, most notably *Microdochium nivale*, *Typhula ishikariensis*, *Typhula incarnata*, and *Sclerotinia borealis* [18]. Additionally, *Leptosphaeria sclerotioides* (syn. *Phoma sclerotioides*), which had previously been proposed as a causal agent of snow mold [21,22,51,52,53], was among the most abundant fungi in plants with severe snow mold lesions in the Middle Volga region, supporting its potential role in disease development during and shortly after plant wintering [22].

In our study, among the most well-known snow mold agents, only *Microdochium* was detected at sufficient abundance. *Leptosphaeria* was also among the most abundant taxa, supporting prior predictions of its role in snow mold development. In addition, *Herpotrichia* (synonyms: *Racodium*, *Nematostoma*) was detected at high abundance in cereal roots after wintering in our study. Species of this genus have previously been associated with black snow mold in conifers during winter [18,54,55,56,57,58]. However, *Herpotrichia* has not been previously reported in cereal crops. Its identification in cereal roots shortly after wintering establishes it as a novel candidate causal agent of snow mold in these crops, warranting further investigation into its potential to cause damage in winter cereals during overwintering. On average, *Microdochium*, *Leptosphaeria*, and *Herpotrichia* were each represented at roughly 3–5% relative abundance within the studied fungal communities, whereas *Typhula* and *Sclerotinia* were present at very low levels (<0.1%). Consequently, we considered *Microdochium*, *Leptosphaeria*, and *Herpotrichia* as the primary putative candidates responsible for the observed snow mold lesions in winter cereals. However, two of these candidates, *Microdochium* and *Leptosphaeria*, were not enriched in diseased plants compared to healthy ones; moreover, *Leptosphaeria* was more abundant in healthy rye plants than in diseased ones. The only putative candidate snow mold pathogen enriched in diseased plants (rye and wheat) compared to healthy plants was *Herpotrichia*, further highlighting its potential role in winter damage to cereal crops. Nevertheless, differences in *Herpotrichia* abundance between healthy and diseased plants, although statistically significant, were rather modest.

Since differences in the abundance of candidate snow mold pathogens between healthy and diseased plants, if observed, were rather modest, we hypothesized that the presence or absence of disease lesions is more likely associated with differences in the overall microbiome composition, as expressed by α- and β-diversity, than with differences in the abundance of specific phytopathogens. However, although some differences in α- and β-diversity between healthy and diseased plants were observed, these differences were minor and could not reliably distinguish disease status. To date, no clear consensus has emerged regarding the relationship between community diversity and disease manifestation. While some studies suggest that healthy plant microbiomes exhibit higher diversity than those of diseased plants [59], accumulating evidence indicates that disease development may not necessarily reduce, and in some cases may even increase, the diversity of plant-associated microbial communities [60,61,62,63,64,65]. We also observed that between-crop microbiome variability is more pronounced between healthy plants than between diseased ones, suggesting a trend toward increased similarity in microbial communities across different crops following disease development. This trend is inconsistent with the previously proposed Anna Karenina principle, which posits that microbiomes of different crops, in the absence of disease, exhibit greater similarity than those of similar crops under conditions of disease manifestation [66]. However, this principle is not considered universal, as numerous studies (including those on oomycete Albugo-infected Arabidopsis and *Ralstonia solanacearum*-infected tomato) have revealed the opposite trend [8,67].

To identify taxa that contributed most to the small but significant β-diversity differences between healthy and diseased plants, DAT were identified. Most identified DAT exhibited low abundance (<1%) within their respective communities. DAT with higher abundance (above 1%) and enriched in healthy plants consisted of microorganisms known to have plant-beneficial effects: *Cladosporium* and *Streptomyces* (for wheat), *Tetracladium* (for triticale), and *Luteolibacter* and *Janthinobacterium* (for rye).

Although phytopathogenic representatives have been described within the *Cladosporium* genus [68,69,70,71], its members are generally recognized for their beneficial roles, including the repression of phytopathogens [72,73,74,75,76,77]. Similarly, although phytopathogenic *Streptomyces* are known [78,79], none have been documented as cereal parasites; this genus is primarily considered to include plant growth-promoting bacteria (PGPB) and producers of antimicrobial compounds [80,81,82,83]. *Tetracladium* is known for its ecological versatility and is a highly abundant root inhabitant (ranking second in abundance in our study). Additionally, representatives of the genus *Tetracladium* have been shown to inhibit the growth of phytopathogenic fungi and to prime the plant immune system [84,85,86]. *Luteolibacter* and *Janthinobacterium* have been well-documented as plant growth-promoting bacteria [87,88,89]. Although the aforementioned microorganisms warrant consideration for their potential role as microbial markers of snow mold resistance, the degree of their differential abundance between healthy and diseased plants was rather small. Thus, although differential abundance testing identified several candidate taxa that could potentially contribute to the suppression of snow mold, it provided limited evidence to explain the reasons for the differential manifestation of the disease.

We further hypothesized that differential disease manifestation could be explained by differences in microbial interactions, which could be reflected in exploratory cross-domain co-occurrence networks within the communities of healthy and diseased plants. Previous studies have shown that such differences can occur even when only minor distinctions in community composition are observed between healthy and diseased plants [7,90,91]. Differences in microbial interaction networks are often reflected in the topological parameters of these networks, such as modularity, average path length, clustering coefficient, and edge density [6]. When networks exhibit strong topological differences, these parameters usually show coordinated differences across compared samples [92,93]. In our study, however, only individual network topology parameters differed modestly yet significantly between the communities of healthy and diseased plants, and these differences were crop-specific. This suggests that the topology of the interaction networks differed only slightly between healthy and diseased plants. Such specific, modest differences in microbial network topology have also been observed between olives with varying degrees of Verticillium wilt damage [64] and in chili pepper (*Capsicum annuum* L.) plants differentially affected by Fusarium wilt [94].

Marked differences in the communities of healthy and diseased plants were observed in our study with respect to inferred candidate hub taxa, which are key nodes of whole-community-scale microbial interactions and are considered crucial components that determine the properties of microbial communities. Differences in these taxa between healthy and diseased plants may therefore reflect not only a taxonomic shift, but also a reorganization of the core interaction networks within the microbiome. Such reorganization is commonly attributed to the loss of taxa that support a stable plant-associated microbiome and/or the emergence of taxa that are more compatible with the pathogen [6,8,9,10,11]. Within rye and wheat, the hub taxa lists were entirely distinct between healthy and diseased plants. Within triticale, the lists were almost completely different between healthy and diseased plants, with the exception of two shared taxa: *Plantibacter* and *Lapillicoccus*. The hub taxa in diseased plants were entirely crop-specific. Within healthy plants, the hub taxa were almost crop-specific, with two exceptions: *Arthrobacter* was a shared hub between rye and triticale, and *Variovorax* was a shared hub between wheat and triticale. These findings align with previous studies on chili pepper and watermelon differentially affected by Fusarium wilt, which showed a complete mismatch of hub taxa between the microbial communities of healthy and diseased plants, without substantial changes in other parameters such as α- and β-diversity or network topology [94,95].

Among the revealed hub taxa, we highlighted key hub taxa—defined as those forming part of the core microbiome and characterized by a relative abundance above 0.1—as having the greatest expected impact on the community and the host plant’s disease status.

Given the dramatic differences in hub taxa between healthy and diseased plants, we hypothesized that the interaction patterns between the target candidate snow mold pathogens (*Microdochium*, *Leptosphaeria*, and *Herpotrichia*) and other microbial community members, including those belonging to hub taxa and/or DAT, would also differ, forming a basis for differential disease manifestation. To test this hypothesis, we first identified taxa whose abundance correlated with the abundance of at least one of the snow mold pathogens in each of the six datasets (representing three crops, each under two disease statuses). Among these “correlated taxa”, we identified a subset (referred to as “target taxa”) expected to have the strongest influence on snow mold pathogens based on at least one of the following criteria: (1) membership in hub taxa (including key hub taxa); (2) differential abundance between healthy and diseased plants (DAT); (3) a reversal in correlation sign (from positive to negative or vice versa) between the healthy and diseased states (correlation sign reversal (CSR)).

The patterns of differences in target taxa between healthy and diseased plants were crop-specific. In rye, most target taxa were those whose abundance positively correlated with that of snow mold pathogens specifically in diseased plants, but not in healthy ones: the abundance of *Pedobacter* (a key hub) and *Devosia* (a DAT enriched in diseased plants) positively correlated with the abundance of *Microdochium*; the abundance of *Terrabacter* and *Fluviicola* (both hubs) positively correlated with the abundance of *Leptosphaeria*; the abundance of *Oliveonia*, *Dyadobacter* (both DATs enriched in diseased plants), and *Kineosporia* (a key hub) positively correlated with the abundance of *Herpotrichia*. These taxa can be presumed to enhance the harmfulness of snow mold pathogens. An interesting pattern was observed for *Caulobacter*: its abundance was positively correlated with *Herpotrichia* in diseased rye, whereas it was negatively correlated in healthy rye. This phenomenon is defined in our study as a correlation sign reversal (CSR).

Among the putative candidate snow mold pathogens in rye, *Herpotrichia* had the highest number of target taxa exhibiting positive abundance correlations with it within diseased plants. Given this finding and the significantly higher abundance of *Herpotrichia* in diseased rye compared to healthy rye, we implicated this genus as the most likely causal agent of winter damage in rye. In healthy rye, no target taxa showed a positive correlation of abundance with any snow mold pathogen. Herewith, the abundance of two taxa was negatively correlated with that of snow mold pathogens: the aforementioned *Caulobacter* (correlation with the abundance of *Herpotrichia*) and *Mycobacterium*, whose content was negatively correlated with that of *Leptosphaeria*. Therefore, *Mycobacterium* may be presumed to provide some protective effect against *Leptosphaeria* in rye. However, it should be noted that rye is likely to display relative tolerance to *Leptosphaeria*, since the abundance of this pathogen was higher in healthy plants than in diseased plants, suggesting that differential snow mold manifestation in rye is not simply associated with higher *Leptosphaeria* abundance in diseased plants.

Thus, according to the obtained data, it can be presumed that the development of severe snow mold symptoms in rye is driven by the fact that snow mold pathogens interact (positive correlation in abundance) with a number of hub taxa and taxa enriched in diseased plants; in healthy plants, such kinds of positive interactions of snow mold pathogens are not realized, which presumably prevents disease development, even taken that the abundance of snow mold pathogens did not differ (or differed modestly) between healthy and diseased plants.

In triticale, the pattern of differences in target taxa between healthy and diseased plants was the least evident among studied crops. More target taxa were revealed in healthy plants than in diseased plants. Among taxa that can be presumed to restrict disease development, the most likely were bacteria of the *Pseudomonas* genus (a key hub) and fungi of the *Tetracladium* genus (a DAT enriched in healthy plants), where the abundance of both of them negatively correlated with the abundance of snow mold pathogens (*Microdochium* and *Herpotrichia*, respectively) within healthy plants. Additionally, for representatives of both *Tetracladium* and *Pseudomonas*, microorganisms with phytopathogen-restricting properties have been described [26,84,85,86,96].

The most evident pattern of differences in target taxa between healthy and diseased plants was observed in wheat. All except one of the target taxa had correlations in abundance with the abundance of *Herpotrichia*. Importantly, the list of these taxa was identical in healthy and diseased plants, but the signs of all correlations were reversed: the abundance of *Mesorhizobium* and *Streptomyces* was negatively correlated with the abundance of *Herpotrichia* in diseased plants but positively correlated in healthy plants, whereas the abundance of *Microscypha*, *Cryobacterium*, and *Pedobacter* was positively correlated with the abundance of *Herpotrichia* in diseased plants but negatively correlated in healthy plants.

It can be speculated that the negative correlations between the abundances of *Microscypha*, *Cryobacterium*, and *Pedobacter* and that of *Herpotrichia* (observed in healthy plants) reflect antagonistic interactions that suppress disease development, and that the switch of these correlations from negative to positive (observed in diseased plants) contributes to the manifestation of snow mold. This assumption is supported by evidence that representatives of the genus *Pedobacter* have been shown to inhibit the growth of phytopathogenic fungi, prime plant immunity, and suppress disease development [97,98,99,100], whereas representatives of *Cryobacterium*, being highly psychrotolerant, promote plant fitness [101,102,103].

In contrast, the positive correlations between the abundances of *Mesorhizobium* and *Streptomyces* and that of *Herpotrichia* (observed in healthy plants) may reflect the plant’s “cry for help” strategy, in which the host plant recruits beneficial microbes to fight the pathogen under its increasing pressure [104,105]. This phenomenon entails a coordinated increase in the abundance of specific beneficial microbes as phytopathogen load increases, thereby suppressing disease development. The switch of these correlations from positive to negative (observed in diseased plants) suggests a breakdown of this defensive communication, ultimately contributing to the manifestation of snow mold. This hypothesis is supported by evidence that *Mesorhizobium* [106,107], and particularly *Streptomyces* [94,104], have been shown to participate in the plant’s “cry for help” response, actively shaping the microbiome to counteract increasing pathogen pressure.

It remains to be determined whether the aforementioned hypotheses are correct, particularly through targeted isolation and functional testing of the candidate taxa on a broad range of cultivars and across different environmental conditions. It also cannot be excluded that the specific set of target taxa may vary depending on the agrocenosis and the weather conditions of a particular year or that the observed microbiome shifts represent consequences rather than causes of disease development; this context-dependency likewise remains to be established. Nevertheless, our study implicates promising candidates whose interactions with snow mold pathogens may modulate disease severity and therefore merit further detailed investigation. The mechanisms underlying the shift in interaction strategies between these target taxa and snow mold pathogens remain enigmatic. However, it is plausible that the differential interactions between the target taxa and snow mold pathogens are driven by variation in plant colonization—specifically, by the presence of distinct species within the target genera, or by different genotypes within the same species—each of which may employ unique interaction strategies with snow mold pathogens, ultimately resulting in distinct levels of disease manifestation.

## 5. Conclusions

Our findings bolster the link between *Leptosphaeria* and snow mold disease in winter cereals, while offering the first hypothesis that fungi of the genus *Herpotrichia* could be implicated as candidate causal agents of snow mold in winter cereals and therefore deserve further experimental investigation. Variations in snow mold severity defy simple explanations tied solely to pathogen abundance or to overall shifts in microbial community composition. Instead, the most striking difference between healthy and diseased plants emerged in shifts of hub taxa and marked changes in co-occurrence patterns within exploratory cross-domain networks involving snow mold pathogens and other community members, with both the hubs and key pathogen correlations being crop-specific. These insights indicate that snow mold progression depends not just on the presence and critical abundance of primary pathogens but also on their interactions within the plant-associated microbiome. We pinpoint several taxa (*Microscypha*, *Cryobacterium*, *Pedobacter*, *Mesorhizobium*, *Streptomyces*, *Pseudomonas*, *Tetracladium*, *Devosia*, *Terrabacter*, *Fluviicola*, *Oliveonia*, *Dyadobacter*, and *Kineosporia*) as probable influencers of snow mold dynamics, meriting targeted follow-up studies to determine their protective or exacerbating contributions to snow mold severity. Our findings support a paradigm shift in plant disease management strategies—from primarily targeting pathogen elimination toward microbiome stewardship, an approach focused on maintaining beneficial network structures, preventing disruptive hub turnover, and modulating interactions between snow mold pathogens and specific community members.

## Figures and Tables

**Table 1:** Sequencing depth and numbers of the revealed amplicon sequence variants (ASV) and taxa.

	Fungi (ITS2)	Bacteria (16S)
Median number of high-quality reads per sample	41,462	14,053
Number of ASVs ^1^	609	2381
Number of taxa (genus or higher rank)	176	303
Good’s coverage, %	99.98 ± 0.008	99.61 ± 0.13

^1^ Number of ASVs after the removal of the low-abundant ASVs and ASVs corresponding to non-bacterial/non-fungal taxa.

**Table 2:** Taxa whose abundance correlated with at least one of the considered putative snow mold pathogens (*Microdochium*, *Leptosphaeria*, and *Herpotrichia* (grey background)) within root communities from healthy and diseased plants across three winter cereal crops (rye, triticale, and wheat), and additionally meet at least one of the following criteria, as noted in the “Feature” column: (1) belong to hub taxa (Hub), including key hub taxa (K-Hub); (2) exhibit differential abundance between healthy and diseased plants (SE; significantly enriched in diseased plants if “SE” is in the “Diseased” column, or in healthy plants if in the “Healthy” column); or (3) exhibit a reversal of correlation sign between healthy and diseased states (CSR; correlation sign reversal). All listed taxa have a relative abundance greater than 0.1%. Bacterial taxa are indicated by a yellow background, while fungal taxa are shown with a blue background. SCC—Spearman correlation coefficient. Only correlations with r > |0.5| and Benjamini–Hochberg FDR-adjusted *p* < 0.05 were considered.

**Genus**	**SCC**	**Feature**	**Genus**	**SCC**	**Feature**
**Rye**					
**Diseased**			**Healthy**		
***Microdochium***			***Microdochium***		
*Chaetomidium*	−0.77	SE			
*Pedobacter*	0.66	K-Hub			
*Chryseolinea*	−0.64	Hub			
*Devosia*	0.71	SE			
***Leptosphaeria***			***Leptosphaeria***		
*Terrabacter*	0.91	Hub	*Mycobacterium*	−0.65	K-Hub
*Fluviicola*	0.79	Hub			
***Herpotrichia***			***Herpotrichia***		
*Oliveonia*	0.66	SE	*Caulobacter*	−0.67	CSR
*Caulobacter*	0.65	CSR			
*Kineosporia*	0.71	K-Hub			
*Dyadobacter*	0.64	SE			
**Triticale**					
**Diseased**			**Healthy**		
***Microdochium***			***Microdochium***		
*Plantibacter*	0.74	K-Hub	*Leptosphaeria*	−0.70	
*Trematosphaeria*	−0.68	CSR	*Trematosphaeria*	0.64	CSR
			*Mrakia*	0.68	SE
			*Pseudomonas*	−0.85	K-Hub
***Leptosphaeria***			***Leptosphaeria***		
*Acremonium*	−0.68	SE	*Pseudogymnoascus*	0.68	K-Hub
***Herpotrichia***			***Herpotrichia***		
			*Tetracladium*	−0.78	SE
**Wheat**					
**Diseased**			**Healthy**		
***Microdochium***			***Microdochium***		
			*Alternaria*	−0.73	SE
***Herpotrichia***			***Herpotrichia***		
*Microscypha*	0.81	CSR	*Microscypha*	−0.64	CSR
*Cryobacterium*	0.75	CSR	*Cryobacterium*	−0.79	CSR
*Mesorhizobium*	−0.82	CSR	*Mesorhizobium*	0.67	CSR
*Pedobacter*	0.77	CSR	*Pedobacter*	−0.7	CSR
*Streptomyces*	−0.81	CSR	*Streptomyces*	0.89	CSR&SE

## Data Availability

The datasets generated during the current study are available in the NCBI Sequence Read Archive (SRA) repository under BioProject accession PRJNA1380312 (https://www.ncbi.nlm.nih.gov/bioproject/PRJNA1380312) (accessed on 22 December 2025). Raw sequencing reads are deposited as follows: 16S rRNA amplicon data under accessions SRR36460514-SRR36460632, and ITS2 amplicon data under accessions SRR36568975-SRR36569094. The current study analyzes only a subset of samples from BioProject PRJNA1380312, which are listed in Supplementary Table S7. ASV tables, taxonomic annotations, sequences and sample metadata are available on GitHub at https://github.com/ildar-ITS/2026_Healthy_Diseased_16S_ITS2_Roots_W_T_R_microbiome (accessed on 22 December 2025).

## Supplementary Materials

The following supporting information can be downloaded at: https://www.mdpi.com/article/10.3390/jof12070496/s1, Table S1: Number of reads before and after non-target ASV removal; Table S2: Comparison of α-diversity indices (Chao1, Shannon, and Gini–Simpson) in fungal and bacterial root communities of winter cereals (rye, wheat, triticale) with different disease statuses (diseased and healthy); Table S3: Differentially abundant bacterial and fungal taxa in the roots of winter cereal crops (rye, wheat, and triticale) between healthy and diseased plants; Table S4: Major topological properties of co-occurrence networks for combined fungal and bacterial root communities in healthy and diseased plants of winter cereal crops (rye, wheat, and triticale); Table S5: Top 5% hub taxa in combined fungal and bacterial root communities in healthy and diseased plants of winter cereal crops (rye, wheat, and triticale); Table S6: Fungal and bacterial taxa whose relative abundance showed significant correlation (r > |0.5|, Benjamini–Hochberg FDR-adjusted *p* < 0.05) with the abundance of at least one snow mold phytopathogen (*Microdochium*, *Leptosphaeria*, or *Herpotrichia*) in root microbiomes of healthy and diseased winter cereal crops (rye, wheat, and triticale); Table S7: SRA run accessions of root samples used in this study.
